# Association between Adequate Fruit and Vegetable Intake and CVDs-Associated Risk Factors among the Malaysian Adults: Findings from a Nationally Representative Cross-Sectional Study

**DOI:** 10.3390/ijerph19159173

**Published:** 2022-07-27

**Authors:** Lay-Kim Tan, Geok-Pei Lim, Hui-Chin Koo, Muhd-Zulfadli-Hafiz Ismail, Yee-Mang Chan, Wahinuddin Sulaiman, Osman Ali, Chee-Cheong Kee, Mohd-Azahadi Omar

**Affiliations:** 1 Sector for Biostatistics & Data Repository, Office of NIH Manager, National Institutes of Health, Ministry of Health Malaysia, Shah Alam 40170, Selangor, Malaysia; m.zulfadli@moh.gov.my (M.-Z.-H.I.); kee@moh.gov.my (C.-C.K.); drazahadi@moh.gov.my (M.-A.O.); 2 Faculty of Applied Sciences, Tunku Abdul Rahman University College, Kuala Lumpur 53300, Malaysia; geokpei1228@gmail.com (G.-P.L.); koohc@tarc.edu.my (H.-C.K.); 3 Institute for Public Health, National Institutes of Health, Ministry of Health Malaysia, Shah Alam 40170, Selangor, Malaysia; chanyeemang@moh.gov.my; 4 Faculty of Medicine, Universiti Kuala Lumpur Royal College of Medicine Perak, Ipoh 30450, Perak, Malaysia; wahinuddin@unikl.edu.my (W.S.); osmanali@unikl.edu.my (O.A.)

**Keywords:** dietary practice, fruit and vegetable intake, diabetes, hypertension, hypercholesterolemia

## Abstract

This study aimed to investigate the relationship between adequate fruit and vegetable intake, and cardiovascular diseases (CVDs)-associated risk factors (i.e., diabetes, hypertension and hypercholesterolemia) among Malaysian adults without history of chronic diseases. We analyzed the data from 11,172 Malaysian adults (i.e., 5554 male and 5618 female), who participated in the population-based National Health and Morbidity Survey 2015. Multiple logistic regression was employed to determine the relationship between adequate daily intake of fruit and vegetables (i.e., ≥5 servings per day) and undiagnosed diabetes, undiagnosed hypertension, and undiagnosed hypercholesterolemia, after adjustment for sociodemographic characteristics and lifestyle risk factors. The mean age (±SE) of these participants was 40.79 (±0.17) years old. Our data demonstrated an adequate daily intake of fruit and vegetables was inversely associated with undiagnosed hypercholesterolemia (adjusted OR: 0.71; 95% CI: 0.51–0.98). Further analyses demonstrated an inverse association between the adequate daily intake of vegetables alone and undiagnosed hypertension (adjusted OR: 0.71; 95% CI: 0.51–0.98). The findings from this study suggest the need for a holistic public health approach to reinforce public awareness about diet-related diseases, which will eventually aid in the prevention of CVDs among Malaysian adults in the long run.

## 1. Background

Non-communicable diseases (NCDs) are the leading causes of ill health in the world and account for 7 of 10 worldwide deaths, where NCDs disproportionately affect people living in low- and middle-income countries [1,2]. In 2017, 73.4% of global deaths (i.e., 41.1 million) were attributed to NCDs, where cardiovascular diseases (CVDs) accounted for the most NCDs-related deaths (17.8 million) [3]. The impact of NCDs in populations extends beyond ill-health and mortality, with a steady increasing financial burden on healthcare budgets and the nation’s welfare [4,5]. 

In Malaysia, CVDs remain the principal cause of death from the total medically certified deaths, with a steady increase for a period of eight years (year 2013 to 2020), i.e., from 13.7% to 17.0% [6]. Diabetes, hypercholesterolemia, and hypertension are among the well-established modifiable CVDs-associated risk factors [6]. The latest national health and morbidity survey reported that a total of 3.9 million (18.3%) Malaysian adults were living with diabetes, 6.4 million (30%) with hypertension, and 8 million (38.1%) with hypercholesterolemia [7].

Good dietary practice, including adequate fruit and vegetable intake—at least 2 servings of fruits and at least 3 servings of vegetables per day—is recommended [8] based on the findings where an adequate intake of fruit and vegetables reduces the risk of CVDs through a beneficial combination of micronutrients, antioxidants, phytochemicals, and fiber in these foods [9]. Findings from the global dietary risks for NCDs study showed a poor dietary practice across the globe, where a continuous global trend in the growth of dietary-risk-related NCDs was reported [10]. The authors further reported that dietary-risk-related NCDs accounted for an increase in all-cause deaths from 18.9% to 22.7%, over the past 29 years, and CVDs were identified as the leading cause of dietary-risk-related NCDs death and disability-adjusted life-years, and low intake of fruit and vegetables were among the top 10 leading risks [10]. 

Evidence from the epidemiological studies showed that individuals with an adequate intake of fruit and vegetables were protected from the risk of CVDs and/or metabolic syndromes, including hypertension and hypercholesterolemia [11,12]. However, these were reported in the prevalent NCDs or metabolic syndromes cases. A local study investigated the association between intake of fruit and vegetables patterns and health-related variables, i.e., diabetes, hypertension, and hypercholesterolemia, among Malaysian adults [13]. However, this study focused on the group of prevalent diabetes, hypertension, and hypercholesterolemia, where chances of diet pattern and lifestyle modification are higher after diagnoses. Having said this, a study on the prevalence of adequate fruit and vegetable intake among Malaysian adults who were not aware of having diabetes, hypertension, or hypercholesterolemia, and their relationships, will provide more precise insights on the relationship between adequate fruit and vegetable intake, and these NCDs.

On the other hand, the importance of adequate fruit and vegetable intake to health has drawn public health attention in Malaysia, resulting in the revision of the Malaysian Food Pyramid, where fruit and vegetable categories has moved to level 1 (i.e., pyramid base), indicating more servings of fruit and vegetables are needed on a daily basis [8]. The importance of adequate intake of fruit and vegetables could not be emphasized more. However, the prevalence of inadequate fruit and vegetable intake among Malaysian adults was remarkably high (i.e., 94%), although the horticulture industry is a contributing sector in the Malaysia economy as it supplies fresh fruits and vegetables in the market [7,14].

Taken altogether, there is a need to identify the relationship between adequate fruit and vegetable intake and CVDs-associated risk factors, namely, diabetes, hypertension, and hypercholesterolemia in the Malaysian population. The finding(s) may aid public health efforts by enforcing the local public awareness of interventions and improving dietary practices. This is one of the primary public health preventive efforts that have much promise and will subsequently reduce the burden of diseases. Hence, we aimed to investigate: (i) the prevalence of adequacy of fruit and vegetable intake among Malaysian adults who were not aware of having diabetes (undiagnosed diabetes), hypertension (undiagnosed hypertension), and hypercholesterolemia (undiagnosed hypercholesteremia), and (ii) the relationship between adequate daily intake of fruit and vegetables, and undiagnosed diabetes, undiagnosed hypertension, and undiagnosed hypercholesterolemia, using the data from the nationally representative population-based survey in Malaysia.

## 2. Materials and Methods

### 2.1. Study Design and Sampling

We analyzed the data from the National Health and Morbidity Survey (NHMS) 2015. The NHMS 2015 was a cross-sectional study of morbidity, health status, and health care demands among a nationally representative, non-institutionalized general population of adults in Malaysia for adults aged 18 years and above. Briefly, the NHMS 2015 aimed to provide health-related community-based data and information to support the Ministry of Health Malaysia in reviewing its health priorities, programs strategies, activities, and planning in its allocation of resources [15].

The respondents of NHMS 2015 were selected by means of a stratified (i.e., primary stratum and secondary stratum) two-stage proportionate-to-size cluster sampling design, and to ensure national representativeness. The primary stratum involved all the 13 states and 3 Federal Territories, whilst the second-stage secondary stratum comprised the urban and rural areas from each state. The selection of samples was conducted by the Department of Statistics Malaysia using an updated 2014 sampling frame. The sampling frame was made up of 79,240 adjacent geographical areas called enumeration blocks (EBs), with 7.65 million living quarters (LQs). On average, each EB comprises 80 to 120 living quarters (LQs), with an average population of 500 to 600 people. Using the probability-proportional-to-size sampling technique, a total of 869 EBs (536 urban and 333 rural) were randomly chosen for the NHMS 2015. In each selected EB, a total of 12 LQs were randomly chosen, and all the eligible household members within the selected LQs were invited to participate in this survey. The detailed methodology and sampling design of NHMS 2015 were described elsewhere [15].

### 2.2. Ethical Consideration

All eligible respondents were informed about the study, and informed consent was obtained prior to the interviews. The study has obtained ethical approval from the Medical Research and Ethics Committee, Ministry of Health Malaysia (NMRR-14-1064-21877).

### 2.3. Survey Materials and Data Collection

The detailed survey materials and data collection were published elsewhere. Briefly, data collection was performed from March to June 2015 using the standardized pre-validated structured questionnaires: (i) a face-to-face interview using the e-NHMS 2015 application programmed in a mobile device by trained evaluators and (ii) self-administered interview using a hardcopy questionnaire. 

The clinical assessment of anthropometry (i.e., weight, height and waist circumferences) [16], blood pressure measurement [17], and biochemistry tests (i.e., fasting blood glucose and cholesterol) [18,19] for the respondents were performed by the nurse. Briefly, duplicate readings of the weight and height of the respondents were measured using the validated and calibrated Tanita Personal Scale HD319 and SECA Stadiometer 213, respectively. For field implementation, a standard weight was supplied for each team for standardization during the weight measuring process. Then, triplicate readings of blood pressure were measured using the validated and calibrated Omron Japan Model HEM-907 machine. Finally, the validated PA Cardiocheck was used to assess the level of fasting blood glucose and cholesterol, where one replicate reading for each biochemistry test was taken.

As for the dietary assessment, data were collected on the intakes of fruit, vegetables, and plain water only in the questionnaire [15], and no information for other food intakes was collected in the NHMS 2015.

### 2.4. Study Variables

#### 2.4.1. Independent Variables

Four questions were used to assess the fruit and vegetable intake by the participants within a one week period (seven days)—“(Q1) In a typical week, how many days do you eat fruit?”; “(Q2) On the day that you usually eat fruit, how much did you eat?”; “(Q3) In a typical week, on how many days do you eat vegetables?”; and “(Q4) On the day that you usually eat vegetables, how much did you eat on that day?”. Food photographs were used to assist the participants in recalling the consumed serving size of fruits and vegetables. These photographs depicted a single serving of commonly consumed fruits and vegetables. For example, one cup of papaya or one medium-sized apple was considered as a single serving of fruit, whilst one cup of chopped raw leafy green vegetables or a half cup of other vegetables (cooked or chopped raw) was considered a single serving of vegetables.

To determine the total number of fruit servings per day, responses to Q1 and Q2 were multiplied and then divided by seven (7) to yield the daily average intake. An adequate intake of fruit per day was defined as ≥2 servings per day. The calculation of the total number of vegetable servings per day was carried out likewise, and an adequate intake of vegetable servings per day was defined as ≥3 servings per day. Based on the Malaysian Dietitian Guidelines 2020, the intake of fruit and vegetables was classified as adequate when ≥5 servings of fruit and vegetables (i.e., ≥2 servings of fruit and ≥3 servings of vegetable) were consumed per day; otherwise, it was considered inadequate [8].

#### 2.4.2. Dependent Variables

Undiagnosed diabetes, undiagnosed hypertension, and undiagnosed hypercholesterolemia were identified based on self-reported medical history and the measurement of blood fasting sugar and cholesterol levels, and blood pressure, during recruitment. A respondent was classified as undiagnosed diabetes mellitus when the respondent was not known to have diabetes and had a fasting capillary blood glucose of 6.1 mmol/L or more (or non-fasting blood glucose of more than 11.1 mmol/L) [18]. Meanwhile, a respondent was classified as having undiagnosed hypertension when the respondent was not known to have hypertension and had a systolic blood pressure of 140 mmHg or more and/or diastolic blood pressure of 90 mmHg or more [17]. Lastly, a respondent was classified as having undiagnosed hypercholesterolemia when the respondent was not known to have hypercholesterolemia and had a total blood cholesterol of 5.2 mmol/L or more [19]. All individuals with undiagnosed diabetes, undiagnosed hypertension, and/or undiagnosed hypercholesterolemia self-reported not receiving treatments for diabetes, hypertension, and/or hypercholesterolemia. 

#### 2.4.3. Potential Confounders

In this study, the sociodemographic characteristics (i.e., gender, age, ethnicity, residential area, marital status, educational level, and monthly household income) and lifestyle risk factors (i.e., obesity, alcohol consumption, smoking status, and physical activity) were included as covariates in the regression model. Age was categorized into five groups: (i) 18–29 years, (ii) 30–39 years, (iii) 40–49 years, (iv) 50–59 years, and (v) 60 years and above. The ethnicity of the respondents was classified into five categories: (i) Malay, (ii) Chinese, (iii) Indian, (iv) other *Bumiputera*, and (v) others. As for the educational level, the classification was based on the local educational system, (i) no formal education, (ii) primary education, (iii) secondary education, and (iv) tertiary education.

The total monthly household income was categorized using the Malaysian household income classification published by the Department of Statistics Malaysia as follows: (i) bottom 40% (B40, below RM4850), middle 40% (M40, RM4851–RM10,970), and top 20% (T20, above RM10,971). Then, we converted the Malaysian currency to the global currency, i.e., United States Dollar (USD), as follows: (i) B40: below USD1,151, (ii) M40: USD1152–2605, and (iii) T20: above USD2606 (based on the currency exchange rate as at 5th of April 2022). The total monthly household incomes were reported in USD from here onwards. 

In this study, the data for obesity was generated using the general obesity and central adiposity data. A respondent was defined as overweight/obese when the (i) body mass index (BMI) was 25 and above and/or (ii) the waist circumference was 80 cm and above in women, and 90 cm and above in men [16]. Alcohol consumption status was categorized as (i) never and (ii) ever (current and former drinker), whilst smoking status was categorized as (i) never, (ii) current smoker, and (iii) former smoker. The short version of the International Physical Activity Questionnaire was used to assess the level of physical activity of the respondents. The physical activity was categorized as (i) inactive and (ii) active [20].

### 2.5. Statistical Analyses

There was a total of 19,959 Malaysian adults aged 18 years and above that participated in NHMS 2015. As this study focused primarily on the adequacy of fruit and vegetable intake and its relationship with undiagnosed diabetes, undiagnosed hypertension, and undiagnosed hypercholesterolemia, participants with missing data or medically diagnosed with diabetes, hypertension, and hypercholesterolemia were removed from this study. Participants who were not aware of having more than one health condition (i.e., diabetes, hypertension, and hypercholesterolemia) were also excluded from this study to avoid confounding and mediational effects when accessing the relationship between adequate fruit and vegetable intake, and CVDs-associated risk factors. Thus, the final number of individuals without chronic diseases that were included in the data analysis was 11,172 (Supplementary Figure S1). Of these, 432, 1031, and 3936 individuals were not known to have diabetes, hypertension, and hypercholesterolemia, respectively. Hence, this left a total of 5773 healthy individuals with no known underlying medical conditions (Supplementary Figure S1).

Complex sample analyses were employed that considered the stratified multistage cluster sampling that was applied in the study. The sample was weighted to represent the general population of Malaysian adults aged 18 years and above. Descriptive statistics was performed to illustrate the sociodemographic characteristics, lifestyle risk factors, and health status (i.e., undiagnosed diabetes, hypertension, and hypercholesterolemia) of the respondents, stratified by the adequacy of fruit and vegetable intake. Multivariable logistic regression was performed to investigate the relationship between adequate intake of fruit and/or vegetables and the risk of undiagnosed diabetes, hypertension, and hypercholesterolemia. The adjusted odd ratios (aOR), with the respective 95% confidence intervals (95% CIs), were calculated. A *p*-value of less than 0.05 was considered significant. Then, we accessed the predictive ability of the final model using the classification tables and performed the receiver operating characteristics (ROC) analyses. All statistical analyses were conducted using the IBM Statistical Package of Social Sciences (SPSS) for Windows version 27.0 (IBM Corp., Armonk, NY, USA). 

## 3. Results

Table 1 shows the characteristics of the respondents in the present study. The age of the participants was between 18 and 114 years old, with a mean age (±SE) of 40.79 (±0.17) years old (data not shown). Of the 11,172 respondents, 51.9% were males and 44.2% were aged between 18 and 29 years old. The majority of them were Malays (53.9%), who resided in urban area (77.0%); were married (58.2%); had a secondary level education (52.4%); and reported having a low monthly household income, i.e., total monthly household income below USD1151 (65.9%). In terms of the lifestyle risk factors, more than half of the respondents were overweight/obese (49.7%). Our data further demonstrated that 85.3%, 75.7%, and 67.4% of the total respondents were non-drinkers, non-smokers, and physically active, respectively. Concerning the adequacy of fruit and vegetable intake, a remarkably low proportion of the respondents had an adequate intake (2.8%). We further observed that the proportion of respondents with adequate fruit intake alone (9.7%) was lower than the proportion of respondents with adequate vegetable intake alone (10.9%). As for health status, the proportion of undiagnosed hypercholesterolemia was the highest (37.2%), followed by undiagnosed hypertension (12.9%) and undiagnosed diabetes (5.8%). 

We further investigated the characteristics of the respondents by the adequacy of their fruit and vegetable intake (Table 2). The Pearson’s chi-square test showed that there were significant associations between sex (*p* < 0.05), age group (*p* = 0.03), ethnicity (*p* = 0.01), marital status (*p* = 0.02), obesity (*p* < 0.05), alcohol intake (*p* = 0.02), smoking (*p* < 0.05), and physical activity (*p* = 0.01), with daily consumption of fruit and vegetables. We observed males had a lower prevalence of adequate fruit and vegetable intake (2.2%, 95% CI = 1.7–2.7) than females (3.4%, 95% CI: 2.8–4.1) (*p* < 0.05), and individuals aged 60 years and above had the highest prevalence of adequate intake (4.3%, 95% CI: 2.7–6.5) as compared to those who were younger, i.e., aged between 18 and 29 years old (2.3%, 95% CI = 1.8–2.9). Comparing different ethnic groups, Chinese (4.3%, 95%: 3.0–5.3) had a higher prevalence of adequate fruit and vegetable intake than Malays (2.1%, 95% CI: 1.7–2.7). As for marital status, the prevalence of adequate fruit and vegetable intake among married individuals (3.2%, 95%CI: 2.6–3.8) was higher compared to those who were single (2.1%, 95% CI: 1.6–2.7). We also observed a higher prevalence of adequate fruit and vegetable intake among the overweight individuals (3.3%), ever-drinker (3.9%), non-smokers (3.1%), and individuals having an active physical activity level (3.0%). Although the univariate analysis revealed no significant association between adequate fruit and vegetable intake and CVDs-associated risk factors, i.e., diabetes, hypertension, and hypercholesterolemia, we observed a comparable low prevalence of fruit and vegetables in the undiagnosed diabetes (2.6%), undiagnosed hypertension (2.9%), and undiagnosed hypercholesterolemia (2.5%) groups. 

Findings from our association analyses demonstrated that adequate fruit and vegetable intake was inversely associated with undiagnosed hypercholesterolemia (aOR: 0.71, 95% CI: 0.51–0.98), after adjustment for sociodemographic characteristics and lifestyle risk factors (Table 3). We further observed that females, older age, other Bumiputera, and obesity were independently associated with undiagnosed hypercholesterolemia (Table 3). The assessment of the predictive ability of the final model demonstrated that the correct predicted value from the classification table was 62.1%, whilst ROC analysis showed that the area under curve (AUC) was 0.65 (95% CI: 0.64–0.66, *p* < 0.001). Further association analyses between adequate vegetable intake alone and different health status categories demonstrated that only undiagnosed hypertension was significantly associated with adequate vegetable intake (aOR: 0.71, 95% CI: 0.51–0.98), after adjustment for sociodemographic characteristics and lifestyle risk factors (Table 4). We also observed that females, older age, educational level, monthly household income, and obesity were independently associated with undiagnosed hypertension (Table 4). Then, the assessment of the predictive ability of the final models and our data demonstrated that the correct predicted value from the classification table was 85.9%, whilst ROC analysis showed that the area under curve (AUC) was 0.76 (95% CI: 0.74–0.77, *p* < 0.001) (Table 4). No association was observed between adequate fruit intake alone and undiagnosed diabetes, undiagnosed hypertension, and undiagnosed hypercholesterolemia (Supplementary Table S1). 

## 4. Discussion

This is the first study to report the relationship between adequate fruit and vegetable intake and CVDs-associated risk factors, i.e., diabetes, hypertension, and hypercholesterolemia among Malaysian adults using data from a nationally representative health and morbidity survey, i.e., NHMS 2015. We observed an inverse association between adequate fruit and vegetable intake, and undiagnosed hypercholesterolemia after adjustment for sociodemographic characteristics and lifestyle risk factors. We further observed that adequate vegetable intake alone was inversely associated with undiagnosed hypertension but not with the undiagnosed diabetes and undiagnosed hypercholesterolemia. 

The prevalence of adequate fruit and vegetables reported in this present study among the man and women was lower compared to other published data. For instance, the data from the World Health Survey involving 52 countries (mainly the low- and middle-income countries) showed that, overall, 22.4% of men and 21.6% of women consumed an adequate intake of fruit and vegetables daily [21]. In Malaysia, a recent local study reported that 10.2% of men and 10.8% of women among the B40 Malaysian adults were found to consume adequate daily servings of fruit and vegetables [22]. In this present study, the prevalence of adequate fruit and vegetable intake was low among the men (2.2%) and women (3.3%) who consumed more than five servings of fruit and vegetables daily, indicating poor dietary practices among the respondents. One of the plausible explanations for the observed differences could be due to the potential influence of sociodemographic differences (i.e., cultural habits) in dietary consumption [23], which are not analyzed in this present study. On the other hand, the differences that we observed between our data and the published local data [22] can be explained by the different study populations. In this present study, we utilized data from the nationally representative health and morbidity survey, whilst the published local study utilized data collected from the respondents that were from B40 income group (monthly income below USD1151.00) and resided in the Federal Territory of Kuala Lumpur.

The relationship between adequate fruit and vegetable intake, and undiagnosed hypercholesterolemia observed in this study is in accordance with the published data from other studies. For instance, in a large follow-up study comprising 1020 Chinese, the authors reported the beneficial effect of the intake of greater numbers of fruit and vegetables (mean of 377 g/daily) to prevent multimorbidity, including hypercholesterolemia [24]. Another study comprising 840 Iranians reported the similar finding, where a minimum of four servings of fruit and vegetables daily was inversely related to the total cholesterol count [25]. The relationship could be due to the availability of a wide range of nutrients and different bioactive compounds such as fibers, vitamin C, carotenoids, antioxidants, potassium, flavonoids, and other phytochemicals available in fruit and vegetables that possess cardioprotective effects in reducing the cholesterol level [9,26]. 

A wide range of nutrients and different bioactive compounds in fruit and vegetables was also reported to be responsible for lowering the blood pressure and hence reducing the risk of hypertension. However, we observed an adequate intake of vegetables alone was inversely associated with undiagnosed hypertension, but not the combination of adequate intake of fruit and vegetables. This observation was not a surprise. Evidence from several epidemiological studies investigating the relationship between adequate fruit and vegetable intake, and the risk of hypertension showed that while some studies demonstrated that individuals with an adequate intake of fruit and vegetables have lower hypertension risk [27,28], others showed an adequate intake of either fruit or vegetables alone was inversely associated with hypertension [29,30]. Similarly, the evidence from the epidemiological studies investigating the relationship between the adequate intake of fruit and vegetables and the risk of diabetes was inconclusive [31,32]. Our study found no association between fruit and/or vegetable intake with undiagnosed diabetes, which is in line with a study involving the incident type II diabetes mellitus patients [31]. These inconclusive findings in the hypertension and diabetes populations are pending dietary pattern studies—a dietary analysis method that considers the cumulative and interactive effects among dietary components in relation to diseases. This will provide more insights to determine the causal relationship between adequate fruit and vegetable intake, and the NCDs-associated risk factors, i.e., diabetes and hypertension. 

Empowering the local community with knowledge regarding the negative health outcomes associated with poor dietary practices (i.e., disease–diet relationships) has been the continuous effort of various government agencies. However, the data from the local studies revealed that good knowledge and attitudes on disease–diet relationships were not translated to good dietary practices, regardless of their age and gender [33,34]. This could be also the underlying reason for the observed low prevalence of adequate fruit and vegetable intake among the respondents in this study and is pending further investigation. On the other hand, the inadequate daily intake of fruit and vegetables was shown to be due to poor dietary practices usually established in childhood and that tended to carry into later adulthood [33,35], which suggested childhood was an opportune time for health-promoting interventions that incorporate the role of parents/caregivers as positive role models to instill good dietary practices among children [35,36,37]. 

Another contributing factor to poor dietary intake, including inadequate fruit and vegetable intake, could be that food purchase choices are associated with sociodemographic factors. For example, the individuals in the low socio-economic status group consumed foods of lower nutritional value and had lower-quality diets that generally cost less, compared to their higher socio-economic counterparts who consumed more fruit and vegetables [22,38]; this is pending further analyses in the general Malaysian population. The recently published local data that were restricted to the Federal Territory of Kuala Lumpur showed that a staggering 89.5% of the B40 Malaysian adults were found to not consume adequate daily amounts of fruit and vegetables, and the top three factors affecting food purchase choices among the respondents were price (79.4%), availability (75%), and taste (73%) [22]. In this present study, we observed that females, older age, ethnicity, married individuals, obesity, alcohol-drinkers, never-smokers, and physically active individuals were associated with adequate fruit and vegetable intake. Two of these observed factors (i.e., females and non-smokers) were in-line with other locally published data, where the authors reported that those with longer working hours, higher levels of education, higher income, females, non-smokers, and those from East Malaysia were positively correlated with fruit and vegetable consumption likelihood [39]. 

Taken together, a holistic public intervention framework to encourage an optimum daily intake of fruit and vegetables among Malaysian adults is needed. This framework involves both the public health authorities/government agencies and engagement from the community that aim not only to spread knowledge on the diet–disease relationship but also on attitudes and behavior towards affordable good dietary practices that are important to build healthier dietary patterns, for example, combined efforts from various government and public agencies to reinforce knowledge empowerment about the diet–disease relationship to the public in parallel with the pricing intervention. Studies have demonstrated that subsidizing fruit and vegetable purchases among the low-income households was shown to be effective, i.e., meaningful increases in healthy food purchases, including fruit and vegetables among the populations with low socioeconomic status, were observed [40]. This is an effective public health preventive effort in combating NCDs-associated risk factors in the long run that holds much promise and sequentially reduces the burden of diseases as well as the financial burden on the healthcare system.

## 5. Strengths, Limitations, and Future Work

The strength of this study is that this is a nationally representative health morbidity survey with a large number of respondents that are the Malaysian citizens with no history of chronic diseases. Furthermore, the prevalent cases of diabetes, hypertension, and hypercholesterolemia were excluded from this study, where only individuals who were not aware of having diabetes, hypertension, and hypercholesterolemia were included in the disease groups for the association analysis. Hence, we have eliminated the potential biases, i.e., dietary habit modification after diagnosis, giving more strength to the observed findings in this study. In addition, we have controlled the established potential confounders, including sociodemographic characteristics and lifestyle risk factors in this present study. 

Nevertheless, the findings from our study could not explain the causal relationship as this was an observational cross-sectional study. Additionally, a potential self-reporting bias could arise from the recall period/recall bias. Another identified limitation in this study was that the respondents who were identified as undiagnosed diabetes, hypertension, or hypercholesterolemia are pending confirmation of clinical diagnosis. Furthermore, only total cholesterol count was performed in the participants with the absence of triglycerides, low-density lipoproteins, and high-density lipoproteins measurements, which are important in the clinical diagnosis of hypercholesterolemia. 

On the other hand, all fruits and vegetables were assumed to share the same nutrient properties; however, they do not. The nutritional value of each type of fruit and vegetable differs, and the preparation methods—cooked versus raw—affect the nutritional value. Cooked vegetable intake and raw vegetable intake showed different associations with cardiovascular outcomes, where an inverse association was only observed between raw vegetable intake, and CVDs incidence and mortality [41]. In this current study, we did not access/analyze the dietary patterns nor the eating habits among the respondents, and hence there is an absence of data on the nutritive value of the fruit and vegetables consumed by the respondents, not to mention the preparation method of the vegetables. Of note, we utilized the seven-year-old data from the nationally representative morbidity survey, i.e., NHMS 2015, of which the findings from this present study may not be representative for the Malaysian population to date due to dietary habit and lifestyle changes that may have occurred during the period, especially during the COVID-19 pandemic.

Taken all together, a prospective longitudinal study to investigate the causal relationship between inadequate fruit and vegetable intake and newly diagnosed CVDs-associated factors, such as diabetes, hypertension, and hypercholesterolemia among Malaysian adults, is needed to replicate and validate the findings from this present study. Furthermore, future work incorporating the usage of validated food frequency questionnaire (FFQ) is essential in nutritional epidemiology studies to examine the relationship between dietary and nutritional factors, and disease occurrence at a population level. 

## 6. Conclusions

In conclusion, our findings showed an inverse association between adequate fruit and vegetable intake and undiagnosed hypertension and undiagnosed hypercholesterolemia among Malaysian adults. A holistic public health intervention framework is needed to spread knowledge and improve the attitude and practices on the adequate daily intake of fruit and vegetables among Malaysian adults. This may reinforce public awareness and further aid in the prevention of CVDs-related risk factors, such as hypertension and hypercholesterolemia.

## Figures and Tables

**Table 1:** Characteristics of the adult respondents aged 18 years and above (n = 11,172).

Characteristic	Estimated Population	Count (n)	(%)	95% CI
***Sociodemographic***				
**Gender**				
Male	6,189,813	5554	51.9	50.7–53.1
Female	5,730,425	5618	48.1	46.9–49.3
**Age group (years old)**				
18–29	5,245,002	4048	44.2	42.5–45.5
30–39	3,030,760	2753	25.4	24.1–26.8
40–49	1,841,189	1946	15.4	14.5–16.4
50–59	1,086,667	1349	9.1	8.4–9.9
≥60	716,618	1076	6.0	5.5–6.6
**Ethnicity**				
Malay	6,430,387	7271	53.9	51.0–56.9
Chinese	3,071,241	1890	25.8	23.1–28.6
Indian	819,342	791	6.9	5.8–8.1
Other Bumiputera	1,420,092	1063	11.9	10.3–13.7
Others	179,177	157	1.5	1.0–2.2
**Residential area**				
Urban	9,178,765	6563	77.0	75.7–78.2
Rural	2,741,473	4609	23.0	21.8–24.3
**Marital status**				
Single	4,481,211	3359	37.6	36.1–39.1
Married	6,932,218	7145	58.2	56.6–59.7
Widow/widower/divorcee	506,809	668	4.3	3.8–4.7
**Education level**				
No formal education	335,261	426	2.8	2.5–3.3
Primary	1,510,928	1788	12.8	11.9–13.8
Secondary	6,178,500	5770	52.4	50.9–54.0
Tertiary	3,758,422	3094	31.9	30.3–33.6
**Monthly household income**				
B40	7,856,381	7815	65.9	63.8–67.9
M40	3,158,215	2708	26.5	24.8–28.2
T20	905,643	649	7.6	6.3–9.1
*Lifestyle risk factors*				
**Obesity**				
Normal	5,456,723	4750	50.3	48.8–51.8
Overweight/obese	5,390,481	5427	49.7	48.2–51.2
**Alcohol intake**				
Never	10,157,611	10,012	85.3	83.6–86.8
Ever	1,752,905	1151	14.7	13.2–16.4
**Smoking**				
Never	9,028,405	8500	75.7	74.5–77.0
Current	2,723,487	2514	22.8	21.7–24.1
Former	168,246	157	1.4	1.2–1.7
**Physical activity**				
Inactive	3,845,067	3429	32.6	31.3–34.0
Active	7,934,384	7609	67.4	66.0–68.7
**Vegetable and fruit intake**				
Inadequate	11,592,080	10,844	97.2	96.8–97.6
Adequate	328,159	328	2.8	2.4–3.2
**Fruit intake**				
Inadequate	10,768,135	10,060	90.3	89.5–91.2
Adequate	1,152,104	1112	9.7	8.8–10.5
**Vegetable intake**				
Inadequate	10,618,883	9971	89.1	87.9–90.1
Adequate	1,301,355	1201	10.9	9.9–12.1
***Health status***				
Undiagnosed diabetes	409,791	432	5.8	5.1–6.7
Undiagnosed hypertension	980,404	1031	12.9	11.9–14.0
Undiagnosed hypercholesterolemia	3,920,005	3936	37.2	35.8–38.7

Data source from National Health and Morbidity Survey (NHMS) 2015; B40: bottom 40% (household income below USD1151); M40: middle 40% (household income between USD1152–2605); T20: top 20% (household income above USD2606); and 95% CI: 95% confidence interval.

**Table 2:** Characteristics of the adult respondents aged 18 years and above, by adequacy of fruit and vegetable intake, (n = 11,172).

Characteristic	Adequate (n = 328)		Inadequate (n = 10,844)		*p*-Value *
	Prevalence (%)	95% CI	Prevalence (%)	95% CI	
***Sociodemographic***					
**Sex**					
Male	2.2	1.7–2.7	97.8	97.3–98.3	<0.05
Female	3.4	2.8–4.1	96.6	95.9–97.2	
**Age groups (years)**					
18–29	2.3	1.8–2.9	97.7	97.1–98.2	0.03
30–39	2.6	1.9–3.5	97.4	96.5–98.1	
40–49	3.4	2.5–4.7	96.6	95.3–97.5	
50–59	3.4	2.5–4.7	96.6	95.3–97.5	
≥60	4.3	2.9–6.5	95.7	93.5–97.1	
**Ethnicity**					
Malay	2.1	1.7–2.7	97.9	97.3–98.3	0.01
Chinese	4.0	3.0–5.3	96.0	94.7–97.0	
Indian	3.2	1.9–5.3	96.8	94.7–98.1	
Other Bumiputera	2.9	1.9–4.3	97.1	95.7–98.1	
Others	1.7	0.3–9.0	98.3	91.0-99.7	
**Residential area**					
Urban	2.7	2.2–3.2	97.3	96.8–97.8	0.45
Rural	3.0	2.3–3.9	97.0	96.1–97.7	
**Marital status**					
Single	2.1	1.6–2.7	97.9	97.3–98.4	0.02
Married	3.2	2.6–3.8	96.8	96.2–97.4	
Widow/widower/divorcee	2.8	1.6–5.0	97.2	95.0–98.4	
**Education level**					
No formal education	2.0	0.8–5.0	98.0	95.0–99.2	0.36
Primary	2.6	1.8–3.6	97.4	96.4–98.2	
Secondary	2.6	2.1–3.2	97.4	96–97.9	
Tertiary	3.2	2.5–4.1	96.8	95.9–97.5	
**Monthly household income**					
B40	2.8	2.3–3.2	97.2	96.8–97.7	0.40
M40	3.1	2.4–4.0	96.9	96.0–97.6	
T20	3.2	1.8–5.8	96.8	94.2–98.2	
***Lifestyle risk factors***					
**Obesity**					
Normal	2.1	1.6–2.7	97.3	97.8–97.7	<0.05
Overweight/obese	3.3	2.7–4.0	96.7	96.0–97.3	
**Alcohol intake**					
Never	2.5	2.1–3.0	97.5	97.0–97.9	0.02
Ever	3.9	2.8–5.4	96.1	94.6–97.2	
**Smoking**					
Never	3.1	2.6–3.7	96.9	96.3–97.4	<0.05
Current	1.6	1.1–2.3	98.4	97.7–98.9	
Former	0.2	0.0–1.1	99.8	98.9–100.0	
**Physical activity**					
Inactive	2.0	1.5–2.6	98.0	97.4–98.5	0.01
Active	3.0	2.6–3.6	97.0	96.4–97.4	
***Health status***					
**Undiagnosed diabetes**					
Yes	2.6	1.2–5.7	97.4	94.3–98.8	0.81
No	2.9	2.4–3.5	97.1	96.5–97.6	
**Undiagnosed hypertension**					
Yes	2.9	1.8–4.5	97.1	95.5–98.2	0.98
No	2.9	2.4–3.5	97.1	96.5–97.6	
**Undiagnosed hypercholesterolemia**					
Yes	2.5	2.0–3.1	97.5	96.9–98.0	0.23
No	2.9	2.4–3.5	97.1	96.5–97.6	

* Pearson’s chi-square test was performed. Data source from National Health and Morbidity Survey (NHMS) 2015, B40: bottom 40% (household income below USD1151); M40: middle 40% (household income between USD1152–2605); and T20: top 20% (household income above USD2606).

**Table 3:** Associations between adequate fruit and vegetable intake and undiagnosed diabetes, undiagnosed hypertension, and undiagnosed hypercholesterolemia in Malaysian adults aged 18 years and above.

Variable	Undiagnosed Diabetes		Undiagnosed Hypertension		Undiagnosed Hypercholesterolemia	
	aOR ^¥^	95% CI	aOR ^¥^	95% CI	aOR ^¥^	95% CI
**Fruit and vegetable intake**						
Inadequate	1.00		1.00		1.00	
Adequate	0.99	0.42–2.32	0.82	0.46–1.49	0.71 *	0.51–0.98
Sex						
Male	1.00		1.00		1.00	
Female	0.79	0.56–1.12	0.49 *	0.38–0.62	1.44 *	1.26–1.65
**Age group (years)**						
18–29	1.00		1.00		1.00	
30–39	1.09	0.72–1.66	2.03 *	1.50–2.75	1.91 *	1.62–2.27
40–49	1.44	0.95–2.19	3.03 *	2.20–4.17	2.35 *	1.92–2.87
50–59	1.24	0.76–2.05	3.80 *	2.55–5.67	3.15 *	2.53–3.93
≥60	1.64	0.90–3.01	5.98 *	3.99–8.97	2.10 *	1.59–2.76
**Ethnicity**						
Malay	1.00		1.00		1.00	
Chinese	0.55 *	0.35–0.86	0.86	0.63–1.19	0.83	0.68–1.02
Indian	1.50	0.86–2.61	0.64	0.40–1.02	0.79	0.61–1.03
Other Bumiputera	0.97	0.61–1.52	1.03	0.73–1.43	0.77 *	0.61–0.98
Others	2.16	0.78–5.95	1.15	0.47–2.83	0.93	0.57–1.50
**Residential area**						
Urban	1.00		1.00		1.00	
Rural	0.87	0.61–1.23	1.10	0.87–1.38	0.93	0.80–1.09
**Marital status**						
Single	1.00		1.00		1.00	
Married	1.06	0.74–1.52	0.84	0.63–1.12	1.11	0.94–1.30
Widow/widower/divorcee	1.34	0.65–2.74	1.18	0.75–1.86	1.14	0.85–1.53
**Education level**						
No formal education	1.00		1.00		1.00	
Primary	0.51 *	0.26–0.99	1.00	0.63–1.59	0.98	0.67–1.43
Secondary	0.37 *	0.19–0.71	0.68	0.43–1.08	0.84	0.57–1.24
Tertiary	0.29 *	0.15–0.58	0.54 *	0.33–0.89	0.82	0.55–1.22
**Monthly household income**						
B40	1.00		1.00		1.00	
M40	0.77	0.52–1.15	0.72 *	0.54–0.94	0.96	0.82–1.13
T20	0.53	0.23–1.23	0.74	0.44–1.24	1.02	0.77–1.35
**Obesity**						
Normal	1.00		1.00		1.00	
Obese	1.26	0.94–1.69	2.73	2.19–3.41	1.38 *	1.23–1.56
**Alcohol intake**						
Never	1.00		1.00		1.00	
Ever	0.98	0.58–1.66	1.09	0.79–1.52	1.09	0.89–1.35
**Smoking**						
Never	1.00		1.00		1.00	
Current	1.07	0.73–1.56	0.67	0.52–1.59	1.08	0.92–1.27
Former	0.26	0.06–1.20	0.84	0.42–1.66	0.77	0.48–1.24
**Physical activity**						
Inactive	1.00		1.00		1.00	
Active	1.22	0.90–1.64	1.05	0.85–1.29	1.12	0.99–1.28

Data source from National Health and Morbidity Survey (NHMS) 2015. **^¥^** Multiple logistic regression was performed adjusted for sociodemographic (i.e., gender, age, ethnicity, residential area, marital status, educational level, and household monthly income) and lifestyle risk factors (i.e., obesity, alcohol intake, smoking status, and physical activity). There were no significant interactions among the independent variables. The model prediction value was 62.1% with the area under curve (AUC) value of 0.65 (95% CI : 0.64–0.66, *p* < 0.0001). aOR: adjusted odds ratio; 95% CI: 95% confidence interval; and *: *p* < 0.05.

**Table 4:** Associations between adequate vegetable intake only and undiagnosed diabetes, undiagnosed hypertension, and undiagnosed hypercholesterolemia in Malaysian adults aged 18 years and above.

Variable	Undiagnosed Diabetes		Undiagnosed Hypertension		Undiagnosed Hypercholesterolaemia	
	aOR ^¥^	95% CI	aOR ^¥^	95% CI	aOR ^¥^	95% CI
**Vegetable intake**						
Inadequate	1.00		1.00		1.00	
Adequate	0.86	0.57–1.31	0.71 *	0.51–0.98	0.92	0.76–1.11
Sex						
Male	1.00		1.00		1.00	
Female	0.79	0.56–1.13	0.49 *	0.39–0.63	1.44 *	1.26–1.65
**Age group (years)**						
18–29	1.00		1.00		1.00	
30–39	1.10	0.72–1.67	2.04 *	1.51–2.77	1.92 *	1.62–2.27
40–49	1.44	0.95–2.19	3.03 *	2.19–4.17	2.34 *	1.91–2.86
50–59	1.25	0.76–2.05	3.81 *	2.56–5.67	3.14 *	2.53–3.91
≥60	1.66	0.90–3.04	6.03 *	4.03–9.02	2.07 *	1.58–2.73
**Ethnicity**						
Malay	1.00		1.00		1.00	
Chinese	0.55 *	0.35–0.87	0.88	0.64–1.22	0.83	0.68–1.02
Indian	1.50	0.86–2.60	0.64	0.40–1.02	0.79	0.61–1.03
Other Bumiputera	0.98	0.62–1.55	1.07	0.76–1.50	0.78 *	0.61–0.99
Others	2.17	0.78–6.02	1.15	0.47–2.82	0.93	0.58–1.51
**Residential area**						
Urban	1.00		1.00		1.00	
Rural	0.87	0.62–1.23	1.11	0.88–1.4	0.93	0.8–1.09
**Marital status**						
Single	1.00		1.00		1.00	
Married	1.06	0.73–1.52	0.84	0.63–1.12	1.11	0.94–1.30
Widow/widower/divorcee	1.33	0.64–2.73	1.16	0.73–1.82	1.14	0.85–1.52
**Education level**						
No formal education	1.00		1.00		1.00	
Primary	0.51 *	0.26–0.99	1.00	0.63–1.59	0.98	0.67–1.44
Secondary	0.37 *	0.20–0.71	0.68	0.43–1.08	0.84	0.57–1.24
Tertiary	0.29 *	0.15–0.58	0.54 *	0.33–0.89	0.82	0.55–1.22
**Monthly household income**						
B40	1.00		1.00		1.00	
M40	0.77	0.52–1.15	0.72	0.55–0.95	0.96	0.82–1.13
T20	0.53	0.23–1.22	0.74	0.44–1.25	1.02	0.77–1.35
**Obesity**						
Normal	1.00		1.00		1.00	
Obese	1.26	0.94–1.69	2.75 *	2.20–3.43	1.38 *	1.23–1.56
**Alcohol intake**						
Never	1.00		1.00		1.00	
Ever	0.99	0.59–1.66	1.10	0.80–1.53	1.09	0.89–1.35
**Smoking**						
Never	1.00		1.00		1.00	
Current	1.06	0.73–1.55	0.67 *	0.52–0.86	1.09	0.92–1.28
Former	0.26	0.06–1.19	0.84	0.43–1.67	0.78	0.48–1.25
**Physical activity**						
Inactive	1.00		1.00		1.00	
Active	1.22	0.91–1.65	1.06	0.86–1.30	1.12	0.99–1.27

Data source from National Health and Morbidity Survey (NHMS) 2015. **^¥^** Multiple logistic regression was performed adjusted for sociodemographic (i.e., gender, age, ethnicity, residential area, marital status, educational level, and household monthly income) and lifestyle risk factors (i.e., obesity, alcohol intake, smoking status, and physical activity). There were no significant interactions among the independent variables. The model predicted value was 85.9% with the area under curve (AUC) value of 0.76 (95% CI : 0.74–0.77, *p* < 0.0001). aOR: adjusted odds ratio; 95% CI: 95% confidence interval; and *: *p* < 0.05.

## Data Availability

All the generated data during this study are included in this published article and its Supplementary Information files. For data-protection purposes, the data used for this study are not publicly available but are available from the Sector for Biostatistics and Data Repository, Office of NIH Manager, National Institutes of Health Malaysia upon reasonable request and with permission from the Director General of Ministry of Health Malaysia.

## Supplementary Materials

The following supporting information can be downloaded at: https://www.mdpi.com/article/10.3390/ijerph19159173/s1, Figure S1: The selection of individuals for data analysis; Table S1: Association between adequacy of fruit intake (≥2 servings per day) and risk of newly diagnosed diabetes mellitus, hypertension and hypercholesterolemia in Malaysian adults aged 18 years old and above.
