# Supplementary material for: Association between Adequate Fruit and Vegetable Intake and CVDs-Associated Risk Factors among the Malaysian Adults: Findings from a Nationally Representative Cross-Sectional Study
Source: Int J Environ Res Public Health. 2022 Jul 27;19(15):9173. doi: 10.3390/ijerph19159173 (PMC9368066; doi:10.3390/ijerph19159173)
Supplement: Supplementary file 1 [file ijerph-19-09173-s001.zip › ijerph-1775415-supplementary.pdf]

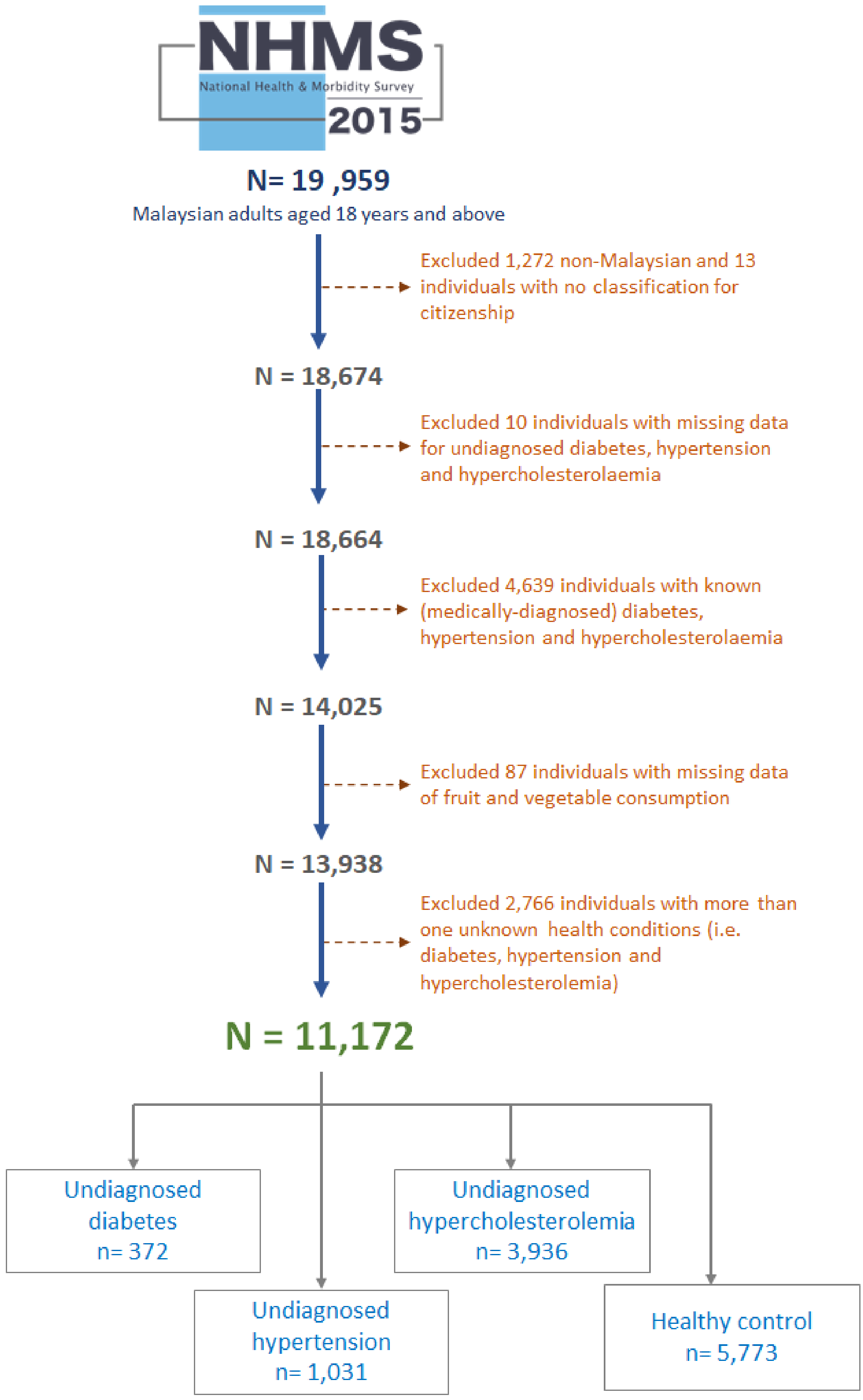

Figure S1. The selection of individuals for data analysis

Table S1. Association between adequacy of fruit intake (>= 2 servings per day) and risk of newly diagnosed diabetes mellitus, hypertension and hypercholesterolaemia in Malaysian adults aged 18 years old and above

Variable	Undiagnosed diabetes (n=432)		Undiagnosed hypertension (n=1,031)		Undiagnosed hypercholesterolaemia (n=3,936)	
	OR	95% CI	OR	95% CI	OR	95% CI
Adjusted Analysis Model 1 (Adjusted for gender and age)						
**Fruit intake**						
Inadequate	1.00		1.00		1.00	
Adequate	1.08	0.68-1.71	0.86	0.63-1.18	0.88	0.73-1.06
**Sex**						
Male	1.00		1.00		1.00	
Female	0.77	0.59-1.00	0.64	0.53-0.77	1.33	1.20-1.48
**Age group (years old)**						
18-29	1.00		1.00		1.00	
30-39	1.13	0.79-1.61	1.87	1.44-2.44	1.97	1.72-2.26
40-49	1.54	1.08-2.20	3.26	2.50-4.26	2.69	2.28-3.16
50-59	1.64	1.04-2.59	4.10	3.02-5.57	3.31	2.78-3.94
≥60	2.12	1.36-3.30	7.71	5.85-10.16	2.16	1.78-2.63
Adjusted Analysis Model 2 (Model 1 + Sociodemographic factors)						
**Fruit intake**						
Inadequate	1.00		1.00		1.00	
Adequate	1.11	0.71-1.74	0.92	0.67 - 1.26	0.90	0.75-1.09
**Sex**						
Male	1.00		1.00		1.00	
Female	0.78	0.59 - 1.04	0.64	0.52 - 0.77	1.33	1.19-1.49
**Age group (years)**						
18-29	1.00		1.00		1.00	
30-39	1.18	0.8 - 1.76	1.99	1.5 - 2.63	1.93	1.65-2.27
40-49	1.66	1.11 - 2.49	3.39	2.52 - 4.57	2.63	2.18-3.18
50-59	1.49	0.89 - 2.48	3.79	2.63 - 5.45	3.14	2.54-3.88
≥60	1.62	0.9 - 2.92	6.07	4.23 - 8.73	1.99	1.53-2.58
**Ethnicity**						
Malay	1.00		1.00		1.00	
Chinese	0.55	0.36 - 0.84	0.79	0.60 - 1.05	0.77	0.64-0.93
Indian	1.65	0.99 - 2.76	0.88	0.58 - 1.33	0.91	0.71-1.17
Other Bumiputera	1.16	0.77 - 1.76	1.17	0.85 - 1.60	0.88	0.70-1.09

Variable	Undiagnosed diabetes (n=432)		Undiagnosed hypertension (n=1,031)		Undiagnosed hypercholesterolaemia (n=3,936)	
	OR	95% CI	OR	95% CI	OR	95% CI
Others	2.54	1.07 - 6.04	1.11	0.47 - 2.62	1.01	0.63-1.61
**Residential area**						
Urban	1.00		1.00		1.00	
Rural	0.88	0.62 - 1.24	1.07	0.86 - 1.33	0.97	0.84-1.12
**Marital status**						
Single	1.00		1.00		1.00	
Married	0.95	0.67 - 1.34	0.97	0.75 - 1.26	0.97	0.84-1.12
Widow/ widower/ divorcee	1.02	0.49 - 2.09	1.16	0.77 - 1.75	0.97	0.84-1.12
No formal education	1.00		1.00		1.00	
Primary	0.63	0.32 - 1.23	0.97	0.75 - 1.26	1.11	0.78-1.58
Secondary	0.46	0.24 - 0.88	1.16	0.77 - 1.75	0.96	0.67-1.37
Tertiary	0.34	0.17 - 0.67	0.97	0.75 - 1.26	0.92	0.64-1.33
**Monthly household income**						
B40	1.00		1.00		1.00	
M40	0.74	0.51 - 1.09	0.80	0.62 - 1.04	0.99	0.85-1.14
T20	0.50	0.22 - 1.15	0.87	0.54 - 1.42	1.04	0.79-1.36
Adjusted Analysis Model 3 (Model 2 + lifestyle factors)						
**Fruit intake**						
Inadequate	1.00		1.00		1.00	
Adequate	1.11	0.69-1.79	0.77	0.54-1.10	0.89	0.73-1.09
**Sex**						
Male	1.00		1.00		1.00	
Female	0.79	0.55-1.12	0.49	0.38-0.62	1.44	1.26-1.64
**Age group (years)**						
18-29	1.00		1.00		1.00	
30-39	1.09	0.72-1.66	2.03	1.50-2.75	1.91	1.61-2.26
40-49	1.44	0.95-2.19	3.03	2.19-4.18	2.34	1.92-2.87
50-59	1.24	0.75-2.05	3.86	2.58-5.76	3.16	2.53-3.93
≥60	1.62	0.88-2.98	6.08	4.06-9.09	2.09	1.59-2.76
**Ethnicity**						
Malay	1.00		1.00		1.00	
Chinese	0.55	0.35-0.86	0.87	0.63-1.2	0.83	0.68-1.01
Indian	1.49	0.86-2.6	0.65	0.41-1.03	0.79	0.61-1.03

Variable	Undiagnosed diabetes (n=432)		Undiagnosed hypertension (n=1,031)		Undiagnosed hypercholesterolaemia (n=3,936)	
	OR	95% CI	OR	95% CI	OR	95% CI
Other Bumiputera	0.96	0.61-1.51	1.04	0.75-1.45	0.77	0.61-0.98
Others	2.13	0.79-5.77	1.18	0.48-2.88	0.94	0.58-1.52
**Residential area**						
Urban	1.00		1.00		1.00	
Rural	0.87	0.61-1.23	1.10	0.87-1.39	0.93	0.8-1.09
**Marital status**						
Single	1.00		1.00		1.00	
Married	1.06	0.74-1.52	0.84	0.63-1.12	1.11	0.94-1.3
Widow/ widower/ divorcee	1.34	0.65-2.73	1.18	0.75-1.86	1.14	0.85-1.53
**Education level**						
No formal education	1.00		1.00		1.00	
Primary	0.51	0.26-0.99	1.01	0.64-1.59	0.98	0.67-1.43
Secondary	0.37	0.19-0.71	0.69	0.43-1.09	0.84	0.57-1.24
Tertiary	0.29	0.14-0.58	0.55	0.34-0.90	0.82	0.55-1.23
**Monthly household income**						
B40	1.00		1.00		1.00	
M40	0.77	0.52-1.14	0.72	0.55-0.94	0.97	0.83-1.13
T20	0.53	0.23-1.22	0.74	0.44-1.25	1.03	0.78-1.36
**Obesity**						
Normal	1.00		1.00		1.00	
Obese	1.25	0.93-1.68	2.75	2.20-3.44	1.39	1.23-1.56
**Alcohol intake**						
Never	1.00		1.00		1.00	
Ever	0.98	0.59-1.65	1.09	0.78-1.50	1.09	0.88-1.34
**Smoking**						
Never	1.00		1.00		1.00	
Current	1.07	0.73-1.56	0.67	0.52-0.86	1.09	0.92-1.28
Former	0.26	0.06-1.20	0.85	0.43-1.67	0.78	0.48-1.25
**Physical activity**						
Inactive	1.00		1.00		1.00	
Active	1.21	0.90-1.63	1.06	0.86-1.3	1.12	0.99-1.28

OR: odds ratio; 95% CI: 95% confidence interval; DM: undiagnosed diabetes; HTN: undiagnosed hypertension; HCL: undiagnosed hypercholesterolaemia; Model 1: adjusted for sex and age; Model 2: Model 1 + socio-demographic variables (ethnicity, residential area, marital status, educational level, household monthly income); Model 3 : Model 2 + lifestyle factors (BMI status, obesity, alcohol consumption, smoking, physical activity)
